# Quality Measures for Gene Expression Biclusters

**DOI:** 10.1371/journal.pone.0115497

**Published:** 2015-03-12

**Authors:** Beatriz Pontes, Ral Girldez, Jess S. Aguilar-Ruiz

**Affiliations:** 1 Department of Computer Languages, University of Seville, Seville, Spain; 2 School of Engineering, Pablo de Olavide University, Seville, Spain; Universidade Federal do Rio Grande do Sul, BRAZIL

## Abstract

An noticeable number of biclustering approaches have been proposed proposed for the study of gene expression data, especially for discovering functionally related gene sets under different subsets of experimental conditions. In this context, recognizing groups of co-expressed or co-regulated genes, that is, genes which follow a similar expression pattern, is one of the main objectives. Due to the problem complexity, heuristic searches are usually used instead of exhaustive algorithms. Furthermore, most of biclustering approaches use a measure or cost function that determines the quality of biclusters. Having a suitable quality metric for bicluster is a critical aspect, not only for guiding the search, but also for establishing a comparison criteria among the results obtained by different biclustering techniques. In this paper, we analyse a large number of existing approaches to quality measures for gene expression biclusters, as well as we present a comparative study of them based on their capability to recognize different expression patterns in biclusters.

## Introduction

DNA microarray technology allows the expression levels of thousands of genes to be measured under several experimental conditions. Usually, the resulting data is organised in a numerical matrix, called an Expression Matrix [1, 2]. Each element of this matrix denotes the numerical expression level of a gene under a certain experimental condition. The analysis of this information can be used to describe interactions between genes, which can in turn enable the discovery or justification of certain biological phenomena [3]. For this reason, there has been great interest in extracting useful knowledge from gene expression data in recent years.

Machine learning techniques have been applied to pursue the goals of understanding regulatory mechanisms, starting from gene expression data [4]. In this regard, one of the most widely studied aims is to find groups of co-expressed genes. If two different genes show similar expression patterns across the conditions, this suggests a common pattern of regulation, possibly reflecting some kind of interaction or relationship between their functions [1]. This correlation between genes can be mathematically represented by two distinct kinds of patterns: shifting and scaling [5]. In this context, clustering techniques aim at finding groups of genes that present a similar variation of expression level under all the experimental conditions. In other words, genes are grouped together according to their expression patterns across the whole set of conditions. This is an important restriction in the use of clustering algorithms, since genes might be co-regulated and co-expressed only under certain experimental conditions, but behave almost independently under other conditions. This is essential for numerous biological problems, such as the analysis of genes contributing to certain diseases, assigning biological functionalities to genes or when the conditions of a microarray are diverse [6]. Thus, clustering should be performed on the two dimensions (genes and conditions) simultaneously. This aspect is covered by biclustering techniques, which have also been widely applied to microarray data [7–10].

Biclustering was introduced in the 1970s by Hartigan [11], although Cheng and Church [12] were the first to apply it to gene expression data analysis. Other names such as co-clustering, bi-dimensional clustering, two-way clustering or subspace clustering often refer to the same problem formulation. In general, biclustering is a much more complex problem than clustering [13]. In fact, it has been proven to be an NP-hard problem [14]. Consequently, most of the proposed biclustering algorithms are based on optimisation procedures such as the search heuristic. Although not all such biclustering approaches base their search on the optimisation of a cost function, most of these algorithms use some kind of measure for assessing or quantifying the quality of biclusters and guiding the search. For such biclustering techniques, the development of both an effective heuristic and a suitable bicluster quality measure is essential for discovering interesting biclusters in an expression matrix. Focusing on the importance of the quality measure, as mentioned previously, finding groups of co-expressed genes, in other words genes that follow a similar expression pattern, is one of the main objectives pursued in the literature. Therefore, the capacity of a measure to recognise different expression patterns in biclusters may be considered to be a suitable criterion for its evaluation.

This paper provides a review of a large number of quality measures for gene expression biclusters as well as a comparative study of them based on their capability to recognise different expression patterns in biclusters. The remainder of this paper is organised as follows: Section *Definitions* presents a unified notation for bicluster representation, a bicluster taxonomy based on the expression patterns exhibited by the genes across the experimental conditions, and a formal description of the shifting and scaling patterns. The third section reviews the different existing evaluation measures for biclusters in the literature. Section *Experimental Analysis and Comparison* discusses the use of the reviewed measures, presents a comparative study based on the experimental analysis of their efficacy for recognising biclusters that follow non-perfect patterns, and studies the relations and dependencies among all the reviewed measures using real data sets. Finally, the main conclusions are summarised in the last section.

## Definitions

Biclusters are represented in the literature in different ways, where genes can be found either in rows or columns, and different names refer the same expression sub-matrix. Here a common notation is defined for biclusters, thereby helping the reader to understand the different approaches taken by authors, even though the original definitions are adapted.

Let, from now on, ℬ be a bicluster consisting of a set *I* of |*I*| genes and a set *J* of |*J*| conditions, in which *b_ij_* refers to the expression level of gene *i* under sample *j*. Then ℬ can be represented as follows:

$$ℬ=\left(\begin{array}{cccc} b_{11} & b_{12} & \ldots & b_{1|J|}\\ b_{21} & b_{22} & \ldots & b_{2|J|}\\ \vdots & \vdots & \ddots & \vdots \\ b_{|I|1} & b_{|I|2} & \ldots & b_{\left|I||J\right|} \end{array}\right)$$

where the gene *g_i_* is the *i^th^* row, e.g., *g_i_*={*b_i1_*,*b_i2_*,…,*b_i|J|_*}, and condition *c_j_* is the *j^th^* column, e.g., *c_j_*={*b_1j_*,*b_2j_*,…,*b_|I|j_*}.

Gene and sample means in biclusters are frequently used in several evaluation measure definitions. These values are represented here as *b_iJ_* and *b_Ij_* referring to the *i* row (gene) and *j* column (sample) means, respectively. Furthermore, the mean of all the expression values in ℬ is referred to as *b_IJ_*. Note that these definitions above may alter the original authors notations in the contributions reviewed in this paper.

### Bicluster Taxonomy based on Gene Expression Patterns

Several types of biclusters have been described and categorised in the literature, depending on the pattern exhibited by the genes across the experimental conditions [15]. For some of them it is possible to represent the values in the bicluster using a formal equation. The following elements are defined thus: *π* represents any constant value for ℬ; *β_i_*(1≤*i*≤|*I*|) and *β_i_*(1≤*j*≤|*J*|) refer to constant values used in additive models for each gene *i* or condition *j*; and *α_i_*,(1≤*i*≤|*I*|) and *α_j_*,(1≤*j*≤|*J*|) correspond to constant values used in multiplicative models for each experimental gene *i* or condition *j*. Hence, biclusters are categorised into the follows types:

- **Constant values**. A bicluster with constant values reveals subsets of genes with similar expression values within a subset of conditions. This situation may be expressed by: *b_ij_* = *π*.
- **Constant values on rows or columns**. A bicluster with constant values in the rows/columns identifies a subset of genes/conditions with similar expression levels across a subset of conditions/genes. Expression levels might therefore vary from gene to gene or from condition to condition. It can also be expressed either in an additive or multiplicative way:
  - Additive: *b_ij_*=*π*+*β_i_*, *b_ij_*=*π*+*β_j_*
  - Multiplicative: *b_ij_*=*π*×*α_i_*, *b_ij_*=*π*×*α_j_*
- **Coherent values on both rows and columns**. This kind of bicluster identifies more complex relationships between genes and conditions, either in an additive or multiplicative way
  - Additive: *b_ij_*=*π*+*β_i_*+*β_j_*
  - Multiplicative: *b_ij_*=*π*×*α_i_*×*α_j_*
- **Coherent evolutions**: Evidence that a subset of genes is up-regulated or down-regulated across a subset of conditions without taking into account their actual expression values. In this situation, data in the bicluster do not follow any mathematical model.

### Shifting and Scaling Expression Patterns

Aguilar-Ruiz [5] presented an in-depth discussion of possible patterns in gene expression data, according to the former definitions. He formally described two kinds of patterns: shifting and scaling patterns. They have been defined using numerical relationships between the values in a bicluster. Several research papers based their principle on the pattern concept in order to mine the data.

A bicluster ℬ follows a *perfect shifting pattern* if its values can be obtained by adding a constant-condition number *β_j_* to a typical value for each gene (*π_i_*). *β_j_* is said to be the *shifting coefficient for condition j*. Graphically, a perfect shifting pattern gives a parallel behaviour of the genes. In this case, the expression values in the bicluster fulfil the following equation:

$$b_{ij}=\pi_{i}+\beta_{j}$$ (1)

Similarly, a bicluster follows a *perfect scaling pattern* pattern by changing the additive value in the former equation to a multiplicative one. This new term *α_j_* is called the *scaling coefficient* and represents a constant value for each condition. In this case, the genes do not follow a parallel tendency. Although the genes present the same behaviour with regard to the regulation, changes are more abrupt for some genes than for others. The following equation defines whether a bicluster follows a perfect scaling pattern or not:

$$b_{ij}=\pi_{i}\times \alpha_{j}$$ (2)

A bicluster may include one of the aforementioned patterns or even both of them, shifting and scaling, at the same time. In fact, it is the most probable case when real data are used. This kind of pattern corresponds to the most general situation that can be described using a mathematical formula, when a bicluster has coherent values on both rows and columns, for the additive and multiplicative model at the same time. When this is the case, it is said that the bicluster follows a *perfect shifting and scaling pattern*, and its values can be represented by this equation:

$$b_{ij}=\pi_{i}\times \alpha_{j}+\beta_{j}$$ (3)

Nevertheless, visually identifying that this bicluster follows a combined pattern is more difficult than finding a single shifting or scaling pattern, since the effects of one influence the other.

## Bicluster Evaluation Measures

Several quality measures for biclusters have been proposed together with different heuristics. Nevertheless, none of the proposed quality measures is able to recognise a perfect shifting and scaling pattern in a bicluster, which is the most general situation and also the most probable when working with gene expression data [16]. This section presents some of the most well-known existing evaluation measures for biclusters.

### Variance (VAR)

Hartigan [11] used bicluster variance in equation 4 as a coherence measure, where the goal of his algorithm was to minimise the sum of bicluster variances.

$$VAR(ℬ)=\sum_{i=1}^{\left|I\right|}\sum_{j=1}^{\left|J\right|}{\left(b_{ij}-b_{IJ}\right)}^{2}$$ (4)

It can be noted that variance only detects constant biclusters. Therefore, biclustering approaches based on variance minimisation methods also include other homogeneity criteria to detect other types of biclusters.

### Mean Squared Residue (MSR)

Cheng and Church [12] were the first to apply biclustering to gene expression data. They introduced one of the most popular biclustering algorithms that combine a greedy search heuristic for finding biclusters with a measure for assessing the quality of such biclusters.

In order to assess the quality of biclusters the algorithm uses the *Mean Squared Residue* (MSR). This measure aims at evaluating the coherence of the genes and conditions of a bicluster *B* consisting of *I* rows and *J* columns. MSR is defined as:

$$MSR(ℬ)=\frac{1}{\left|I|\cdot |J\right|}\sum_{i=1}^{\left|I\right|}\sum_{j=1}^{\left|J\right|}{\left(b_{ij}-b_{iJ}-b_{Ij}+b_{IJ}\right)}^{2}$$ (5)

The lower the mean squared residue, the stronger the coherence exhibited by the bicluster, and the better its quality. If a bicluster has a mean squared residue lower than a given value *δ*, then it is called a *δ*-bicluster.

If a bicluster has a MSR equal to zero, it means that its genes fluctuate in exactly the same way under the subset of experimental conditions, and thus it can be considered a perfect bicluster. Nevertheless, MSR has been proven to be inefficient for finding certain types of biclusters in microarray data, especially when they present strong scaling tendencies. Aguilar-Ruiz [5] proved that $MSR(ℬ)=\sigma_{\alpha }^{2}\sigma_{\pi }^{2}$. That is an important result, because it demonstrates that the quality of a bicluster based on the MSR depends on the product of the scaling coefficient *α_j_* variance ($\sigma_{\alpha }^{2}$ ) and the typical *π_i_* values variance ($\sigma_{\pi }^{2}$), and it does not depend at all on the shifting coefficient *β_j_*. In other words, MSR depends on the scaling variance and not on the shifting variance. This fact is very important, since a high scaling variance would lead us to miss those biclusters that provide a high MSR. In fact, MSR is only able to capture shifting tendencies within the data [17].

### Scaling Mean Squared Residue (SMSR)

Mukhopadhyay et al. [18] have developed an evaluation measure for biclusters that is able to recognise scaling patterns. In their work, they analyse the reasons why MSR is able to recognise shifting patterns in biclusters but not scaling patterns. Using the mathematical formula for scaling patterns, they define a metric that is then proved to identify scaling patterns. This new measure is called SMSR, from *Scaling MSR*, and it is shown in equation 6. Nevertheless, SMSR is not capable of identifying shifting patterns.

$$SMSR(ℬ)=\frac{1}{\left|I|\cdot |J\right|}\sum_{i=1}^{\left|I\right|}\sum_{j=1}^{\left|J\right|}\frac{{\left(b_{iJ}\times b_{I\;j}-b_{i\;j}\times b_{I\;J}\right)}^{2}}{b_{iJ}^{2}\times b_{Ij}^{2}}$$ (6)

### Relevance Index (RI)

Yip et al. [19] proposed an evaluation metric that differs slightly from MSR, in which the quality of a bicluster is measured as the sum of the relevance indices of the columns. *Relevance index R_Ij_* for column *j*∈*J* is defined as:

$$R_{Ij}=1-\frac{\sigma_{\mathrm{Ij}}^{2}}{\sigma j^{2}}$$ (7)

where $\sigma_{\mathrm{Ij}}^{2}$ (local variance) and $\sigma_{\cdot j}^{2}$ (global variance) are the variance of the values in column *j* for the bicluster and the whole data set, respectively. Note that the index gives a high value when the local variance is small compared to the global variance, so the relevance index for a column is maximised if its local variance is zero, provided that the global variance is not. Based on this relevance index, the quality of a cluster is measured as the sum of the index values of all the selected conditions.

Due to the nature of the evaluation, the only bicluster patterns that maximise the quality are constant biclusters (either on rows or on columns).

### Correlation-based Measures

Correlation coefficients have been used extensively in different kinds of microarray analyses, such as clustering. These statistics measure the overall similarity of the genes without placing any emphasis on specific magnitudes, also taking into account negative correlations as well as positive. This subsection reviews the correlation-based approaches proposed for bicluster evaluation.

**Pearson’s Correlation Coefficient (PCC)**. PCC between two variables is defined as the covariance of the two variables divided by the product of their standard deviations. It is a measure of the linear dependency between two variables, and gives a value between +1 and −1, both inclusive. A value of 1 implies that a linear equation describes the relationship between the two variables perfectly (positive correlation). A value of −1 implies that all data points lie on a line for which one variable decreases as the other increases (negative correlation). A value of 0 implies that there is no linear correlation between the variables.

PCC is a very effective metric to quantify co-regulation between pairs of genes [20], and it allows both shifting and scaling patterns to be captured that would be separately identified by additive and multiplicative models, respectively. Nevertheless, PCC is not effective for recognising constant biclusters or constant row patterns, since these kinds of patterns would make the denominator zero.

PCC between two rows (genes) *i_1_*,*i_2_*∈*I* with respect to the columns (conditions) *j*∈*J* is defined as:

$$PCC(i_{1},i_{2})=\frac{\sum_{j=1}^{\left|J\right|}\left(b_{i_{1}j}-b_{i_{1}J})(b_{i_{2}j}-b_{i_{2}J}\right)}{\sqrt{\sum_{j=1}^{\left|J\right|}{\left(b_{i_{1}j}-b_{i_{1}J}\right)}^{2}\sum_{j=1}^{\left|J\right|}{\left(b_{i_{2}j}-b_{i_{2}J}\right)}^{2}}}$$ (8)

where *b*_*i*_1_*j*_ and *b*_*i*_2_*j*_ denote the elements in rows *i*_1_,*i*_2_ and column *j*, and *b*_*i*_1_*J*_, *b*_*i*_2_*J*_ represent the means of rows *i*_1_ and *i*_2_, respectively.

PCC quantifies coherences between pairs of genes. Therefore, in order to measure bicluster coherence, one has to compute all pairwise PCC values between the rows in the same bicluster. Another challenge is that PCC is only meaningful to measure coherence between rows but is too restrictive if it is used to measure coherence between columns simultaneously. PCC has been used as such (between rows) in some research [20, 21], as well as in [20, 21], where the authors define a PCC-based measure called **Average Correlation (AC)**, in the form of equation 9:

$$AC(ℬ)=\frac{\sum_{i_{1}=1}^{|I|-1}\sum_{i_{2}=i_{1}+1}^{\left|I\right|}PCC(i_{1},i_{2})}{\left(\begin{array}{c} \left|I\right|\\ 2 \end{array}\right)}$$ (9)

In order to use PCC for measuring coherence between columns as well, and to overcome the issue of restrictiveness, both Yang et al. [23] and Teng and Chan [24] have defined a PCC-based evaluation measure for bicluster evaluation in terms of improved correlation, either on rows or columns, as defined in the following sections.

**Sub-Matrix Correlation Score (SCS)**. Yang et al. [23] used the *Pearson correlation score* as the basis to define their measure, assuming that a perfect correlated pattern satisfies perfect linear correlation on row and on column vectors. *Scores correlations* for rows and columns are first defined as in equations 10 and 11:

$$S_{row}=\text{min}{\;}_{i_{1}\epsilon I}(S_{i_{1}J}),S_{i_{1}J}=1-\frac{\sum_{i_{2}\neq i_{1},i_{2}\epsilon I}^{}\left|cor(x_{i_{1}J},x_{i_{2}J})\right|}{|I|-1}$$ (10)

$$S_{col}=\text{min}{\;}_{j_{1}\epsilon J}(S_{Ij_{1}}),S_{Ij_{1}}=1-\frac{\sum_{j_{2}\neq j_{1},j_{2}\epsilon J}^{}\left|cor(x_{Ij_{1}},x_{Ij_{2}})\right|}{|J|-1}$$ (11)

where *cor*(*x_i_1_J_*,*x_i_2_J_*) and *cor*(*x_Ij_1__*,*x_Ij_2__*) represent the PCC of any pair of genes or conditions in the bicluster, respectively. *S_row_* score reflects the correlation degree on the rows of the bicluster, while *S_col_* reflects the correlation degree on columns, where both definitions are asymmetric. The *sub-matrix correlation score* is defined as the minimum value of these two scores:

$$S(ℬ)=min(S_{row}(I,J),S_{col}(I,J))$$ (12)

Since absolute values are used in *S_row_* and *S_col_* definitions, a perfect correlated bicluster will satisfy *S*(*I,J*) = 0, meaning that the rows or columns of the bicluster are perfectly linearly correlated. Yang et al. [23] also defined a *δ – corbicluster* as a bicluster that satisfies *S*(*I,J*) < *δ*, for some *δ* > 0.

**Average Correlation Value (ACV)**. ACV was proposed by Teng and Chan [24] to evaluate the homogeneity of a bicluster or a data matrix in the following way:

$$ACV(ℬ)=\text{max}\left\{\frac{\sum_{i_{1}=1}^{\left|I\right|}\sum_{i_{2}=1}^{\left|I\right|}|r\_row_{i_{1}i_{2}}|-|I|}{\left|I{|}^{2}-|I\right|},\frac{\sum_{j_{1}=1}^{\left|J\right|}\sum_{j_{2}=1}^{\left|J\right|}|r\_col_{j_{1}j_{2}}|-|J|}{\left|J{|}^{2}-|J\right|}\right\}$$ (13)

where *r_row_i_1_i_2__*,*r_col_j_1_j_2__* refer to the correlation between any pair of rows *i*_1_,*i*_2_ or columns *j*_1_,*j*_2_, according to the *Pearson coefficient*.

*ACV*(ℬ) has been proven to be in the interval [0,1], where a value equal to 1 means that the rows or columns of the bicluster are highly co-expressed, while a low ACV means that neither the conditions nor the genes are similar. Therefore, higher values of ACV are preferred.

The authors proved in their work that ACV always gives the desirable value for both the additive and the multiplicative model, contrary to MSR. They also performed a study on both ACV and MSR behaviours in seven different types of biclusters.

Although ACV is presented as the criterion to evaluate biclusters, it has not been used to guide the search in their algorithm. Instead, the algorithm is based on the use of a weighted correlation coefficient, a variation of the one proposed by Bland and Altman [25], in which weight factors are assigned to the different features (genes or samples) according to their importance. This way, more important features will have a greater impact than others.

**Average Spearman’s Rho (ASR)**. ASR was first proposed by Ayadi et al. [26] and is based on the use of *Spearman’s rank correlation*, which measures the statistical dependency between two variables, assessing how well their relationship can be described using a monotonic function. Its value varies from −1 to +1, with one of these two values occurring when the two variables being compared are monotonically related, even if their relationship is not linear.

The most important difference between Spearman and Pearson correlations is that Pearson assesses linear relationships, while Spearman assesses monotonic relationships. Hence, the Spearman correlation is less sensitive to strong outliers that are in the tails of both samples. Nevertheless, when the data are roughly elliptically distributed and there are no prominent outliers, the Spearman and Pearson correlations give similar values.

ASR is computed as in equation 14 and outputs a value in the interval [−1,1], where a high/low value close to 1/−1 indicates that the genes or conditions of the bicluster are strongly correlated, either positively or negatively, respectively.

$$ASR(ℬ)=2\times \text{max}\left\{\frac{\sum_{i\epsilon I}^{}\sum_{j\geq i+1,j\epsilon I}^{}\;\rho_{ij}}{\left|I|(|I|-1\right)},\frac{\sum_{k\epsilon J}^{}\sum_{l\geq k+1,l\epsilon J}^{}\;\rho_{kl}}{\left|J|(|J|-1\right)}\right\}$$ (14)

where *ρ_ij_,ρ_kl_* refer to the Spearman correlation between two genes or conditions, respectively.

**Spearman’s Biclustering Measure (SBM)**. SBM has been recently proposed by Flores et al. [27], highlighting its ability to recognise complex coherence patterns in biclusters, such as shifting and scaling, and also negative correlations.

The computation of this coefficient is accomplished in two steps. First, the data is converted into ranks, in order to compute the Spearman coefficient, denoted as *r_x,y_* for any pair of genes or conditions. Using these coefficients, SBM is defined as follows:

$$SBM(B_{ij})=\alpha (B_{ij})\times r_{B_{IJ}}^{\bar{G}}\times \beta (B_{ij})\times r_{B_{IJ}}^{\bar{C}}$$ (15)

where $r_{B_{IJ}}^{\bar{G}}$ and $r_{B_{IJ}}^{\bar{C}}$ denote the summarised expression of the trends observed in the genes and conditions of the bicluster, respectively. They are computed as in equation 16:

$$r_{B_{IJ}}^{\bar{G}}=\frac{2}{\left|I|\times (|I|-1\right)}\times \sum_{i=1}^{\left|I\right|}\sum_{i^{\prime }=i+1}^{\left|I\right|}|r_{ii^{\prime }}^{G}|\;,\;r_{B_{IJ}}^{\bar{C}}=\frac{2}{\left|J|\times (|J|-1\right)}\times \sum_{j=1}^{\left|J\right|}\sum_{j^{\prime }=j+1}^{\left|J\right|}|r_{jj^{\prime }}^{C}|$$ (16)

where $\left|r_{ii^{\prime }}^{G}\right|$ and $\left|r_{jj^{\prime }}^{C}\right|$ correspond to the absolute values of the Spearman’s correlation coefficient between a pair of genes *i,i′* and a pair of conditions *j,j′*, respectively.

Terms *α*(*B_ij_*) and *β*(*B_ij_*) in equation 15 represent the reliability coefficients, and they weight the influence of the trends observed in both genes and conditions, respectively. The value of *β* is set to one due to its high reliability, since it is computed from hundreds of genes. The value of *α*, on the other hand, is associated with the conditions set and therefore has a very low reliability. Its value is computed using the number of samples, in such a way that its value is set to one or $\frac{\left|J\right|}{M}$, depending on the comparison of |*J*| to a certain *threshold*. According to the authors, this *threshold* is selected based on experimentation, with its default value set to nine.

SBM needs to be maximised in order to increase bicluster quality, although no range of values is specified. This means that its value may vary from one dataset to another, even when a bicluster follows a perfect pattern.

### Standardisation-based Measures

One important observation that can be extracted from an analysis of biclusters is that the range of expression values assumed by genes can vary substantially depending on the specific microarray taken as input. Therefore, in order to make an appropriate comparison between each gene and the pattern, a previous standardisation process of the bicluster would enable the expression levels to be scaled to a common range. This mechanism would also be responsible for softening gene behaviour, since the most important aspect is to characterise its tendency rather than its numerical values.

Gene standardisation of a bicluster B corresponds to the standardised bicluster $\hat{B}$, whose element bij^ are obtained as follows:

bij^=bij−μgiσgi,1≤i≤|I|,1≤j≤|J| (17)

where *σ_g_i__* is the standard deviation of all the expression values of gene *i* and *μ_g_i__* is the mean of row *i* in ℬ.

By means of standardisation, two distinct tasks are carried out. The first one is to shift all the genes to a similar range of values (near 0 in this case). The second one is to homogenise the expression values for each gene, modifying in this way their values under all the conditions, and smoothing their graphical representation, due to the correction of the global scaling factor in the denominator.

Three different measures have been defined in the literature, making use of a standardisation process for bicluster evaluation. Two of them (MSA and VE) are based on gene standardisation, while the other one relies on a condition standardisation, as detailed below.

**Maximal Standard Area (MSA)**. The idea behind MSA [28] is to measure the area of the region between the maximum and minimum values of expression levels that genes assume under the conditions contained in the bicluster. Thus, what it is measured is the area depicted by the maximal fluctuation of expression levels for each experimental condition. For each condition, the minimum and maximum values of expression level for all the genes contained in the bicluster are taken. These pairs of values define a band across all the conditions in the bicluster, and the area of this band is, therefore, the measure MSA.

Upper and lower bounds of a bicluster for each condition *j* are defined as *M_j_*(ℬ) and *m_j_*(ℬ) respectively, in the following way:

$$M_{j}(ℬ)=max_{i}\;b_{ij},\forall i;m_{j}(ℬ)=min_{i}\;b_{ij},\forall i$$ (18)

Using these bounds, MSA is defined as the area delimited by the bounds for each condition in the standardised bicluster:

$$MSA(ℬ)=\sum_{j=1}^{|J|-1}\left|\frac{M_{j}(\hat{ℬ})-m_{j}(\hat{ℬ})+M_{j+1}(\hat{ℬ})-m_{j+1}(\hat{ℬ})}{2}\right|$$ (19)

If the genes of a bicluster have a perfectly coherent trend, then MSA(ℬ) is equal to zero. On the contrary, MSA will assume higher values when the genes are less correlated with each other, due to the fact $M(\hat{ℬ})$ and $m(\hat{ℬ})$ are more distant from each other.

**Virtual Error (VE)**. VE [29] follows similar assumptions regarding the standardisation process within the evaluation as MSA. The basic idea behind VE is to measure how genes follow the general tendency within the bicluster. In order to catch this general tendency of the genes across the conditions contained in the bicluster, a new *virtual* row is calculated from the genes of the bicluster, called *virtual pattern* or *virtual gene ρ*. Each element *ρ_j_* of *ρ* is computed as the mean of the *j^th^* column or experimental condition, as in the equation:

$$\rho_{j}=\frac{1}{\left|I\right|}\sum_{i=1}^{\left|I\right|}b_{ij}$$ (20)

Once the virtual gene *ρ* has been computed, and in order to assess how well a specific gene *g_i_* of the bicluster follows the general tendency, VE computes the differences between the expression level values of *g_i_* and the values of *ρ*, for each experimental condition of the bicluster. These differences are computed using the standardised genes in the bicluster and also the standardised virtual gene ${\hat{\rho }}_{j}$:

$$VE(ℬ)=\frac{1}{\left|I|\cdot |J\right|}\sum_{i=1}^{\left|I\right|}\sum_{j=1}^{\left|J\right|}|{\hat{b}}_{ij}-{\hat{\rho }}_{j}|$$ (21)

VE computes the differences between the real genes and the virtual one, once they have been standardised. Therefore, the more similar the genes are, the lower the value for VE. In fact, VE is zero for those biclusters following either a perfect shifting or scaling pattern. Nevertheless, VE cannot be proven to recognise both kinds of patterns simultaneously.

**Transposed Virtual Error (VE^*t*^)**. VE^*t*^ [30] is computed similarly to VE but considering the transposed bicluster. The idea here is to create the virtual pattern in the condition dimension, which is termed *virtual condition*n. Afterwards, differences between the standardised values for every condition and the standardised virtual condition are measured in the same way as in VE.

This way, the virtual condition is computed as in:

$$\rho_{i}=\frac{1}{\left|J\right|}\sum_{j=1}^{\left|J\right|}b_{ij}$$ (22)

and the VE^*t*^ value for bicluster ℬ is obtained using the standardised virtual condition $\hat{\rho }$ together with the standardised bicluster by samples:

VEt(ℬ)=1|I|⋅|J|∑i=1i=|I|∑j=1j=|J||bij^−ρ^i| (23)

VE^*t*^ has been proven to be zero for biclusters with perfect shifting, scaling or combined patterns. Therefore, it is efficient to recognise both shifting and scaling patterns in biclusters either simultaneously or independently.

### Similarity Score

Liu and Wang [31] proposed the use of a *similarity score* between two genes and also a similarity score for a sub-matrix. The first one is used when the reference gene is known in advance. When it is not known, a number of genes might also be randomly selected as the reference genes.

**Similarity Score Between Genes**. The similarity score between two genes (gene *i* and a reference gene i*) under condition *j* is computed as in equation 24:

$$s_{ij}=\{\begin{array}{ll} 0 & \;\mathrm{if}\;d_{ij}>\alpha \cdot d_{avg}\\ 1-\frac{d_{ij}}{\alpha \cdot d_{avg}}+\beta & \;\mathrm{otherwise}\; \end{array}$$ (24)

where *d_avg_* is defined as the average distance value of all the elements in the expression matrix:

$$d_{avg}=\frac{\sum_{i\epsilon I,j\epsilon J}^{}\;d_{ij}}{\left|I||J\right|}$$ (25)

and *d_ij_* is the absolute value of the expression difference between the gene *i* and the reference gene i* for condition *j* in the expression matrix *a*:

$$d_{ij}=|a_{ij}-a_{i*j}|$$ (26)

*α*⋅*d_avg_* is used as a threshold to ignore elements with a large *d_ij_*, in order to find constant biclusters, and *β* is the bonus for small *d_ij_*. This way, *β* enlarges the similarity score for small *d_ij_* and ignores *d_ij_* greater than the threshold.

According to equation 24, the value of *s_ij_* will always be greater than or equal to 0, with 0 being its worst value of similarity.

**Similarity Score for a Bicluster (SS)**. For each row *iϵI*, the similarity score of row *i* in ℬ is:

$$s(i,J)=\sum_{j\epsilon J}s_{ij}$$ (27)

Similarly, for each column *jϵJ*, the similarity score in B corresponds to:

$$s(I,j)=\sum_{i\epsilon I}s_{ij}$$ (28)

Using these equations, the similarity score for a bicluster is computed as the minimum value of the similarity scores of both genes and conditions in the bicluster:

$$s(ℬ)=s(I,J)=\text{min}\{{\text{min}}_{i\epsilon I}\;s(i,J),\min{\;}_{j\epsilon J}\;s(I,j)\}$$ (29)

The goal when looking for biclusters is to find sub-matrices with higher values for the similarity score. In order to improve the quality of the output, Liu and Wang [31] also introduced the *average similarity score* as a second criterion. It consists of the average of all the similarity values of equation 24 for all the elements in the bicluster.

Although the type of bicluster found using the similarity score depends on the values for the different thresholds (*a, β*, and *γ* for the average), only constant and additive biclusters are recognised.

### Experimental Analysis and Comparison

This section discusses the use of the different measures presented above for bicluster evaluation. In particular, the values of each metric are examined along with their variations for biclusters in which the presence of patterns is not perfect. That is, when the tendency of the data in a bicluster is similar to a perfect pattern but does not completely match with the equations in section *Bicluster Taxonomy based on Gene Expression Patterns*.

In order to ascertain the metrics behaviour whenever a bicluster does not follow a perfect pattern, an additive term *ε_ij_* is added to the combined pattern equation. The meaning of this new term corresponds to the error made in the assumption that the bicluster can be represented by a perfect combined pattern:

$$b_{ij}=\pi_{i}\times \alpha_{j}+\beta_{j}+\varepsilon_{ij}$$ (30)

It is possible therefore to study and compare the variations produced in the different evaluation measures depending on the values of *ε_ij_*. Nevertheless, it is not so simple due to the large number of situations depending on the distribution and the magnitude of the *ε_ij_* values in the data matrix. In two specific situations the value of the metrics will not be affected when the errors could be included in equation 30 above. These two cases correspond to those in which *ε_ij_* values are either a constant or constants per condition (columns). In both cases it is possible to eliminate the term *ε_ij_* from the equation, since it can be considered a part of *β_j_*.

Nevertheless, the cases in which *ε_ij_* must be included in the perfect pattern equation are very difficult to study analytically. For this reason, a test has been performed to verify the tendency of the metric values with regard to error values. This test entails adding random errors to a synthetic base bicluster with perfect shifting and/or scaling tendencies. Specifically, 100 synthetic biclusters have been generated adding random errors to the base bicluster, and this process has been repeated 200 different times, varying the amplitude of the errors from one time to another. First, both positive and negative errors were added in the range of [−0.05,0.05], obtaining 100 different biclusters. Then the amplitude was iteratively increased by 0.1 and the process repeated (the next range would be [−0.1,0.1]). Hence, the amplitude of the inserted errors varied from 0.1 to 20, where the maximum amplitude corresponded to range [−10,10]. The whole process produced 200 sets of 100 biclusters with both positive and negative errors drawn from a uniform distribution corresponding to the ranges. Note that the type of error values was a double type. This introduces more diversity in the distribution of the error data.

Within the process, each bicluster produced was evaluated using the majority of the metrics reviewed in section *Bicluster Evaluation Measures*. The Relevance Index (RI) has been excluded from this study in and the Similarity Score in subsection *Similarity Score*, since both of them require additional information apart from the bicluster to be computed. The first one, RI, makes use of the data in the whole input data matrix, while the second one, Similarity Score, requires an input reference gene to calculate the differences with the genes in the bicluster. For the rest of the metrics, the mean of each measures values has been obtained and represented for each group of 100 biclusters with the same range of errors. The base bicluster consists of a synthetic bicluster made up of 12 genes and 7 conditions, where the *π* values have been randomly obtained, varying from −15 to 20. The experimentation has been carried out three times, once for each type of pattern: shifting, scaling and combined (simultaneous shifting and scaling), where the *a* values vary from 6 to 22 (their values have been set to 1 for shifting tendencies), and the *β* values range from 50 to 175 (analogously, their values have been set to 0 for scaling tendencies). All of them also contain negative correlations in their simulated expression levels.

**Figure 1:**
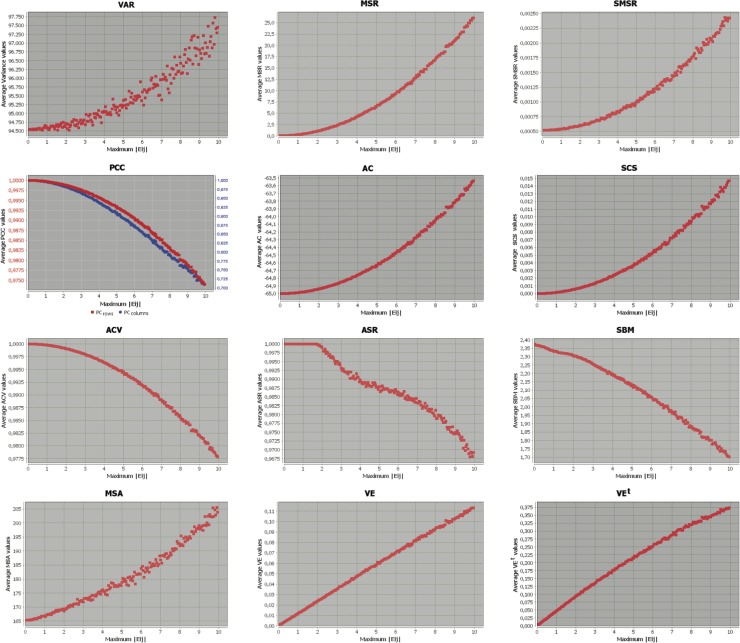
Evaluation measures evolution from a shifting base bicluster with induced errors.

**Figure 2:**
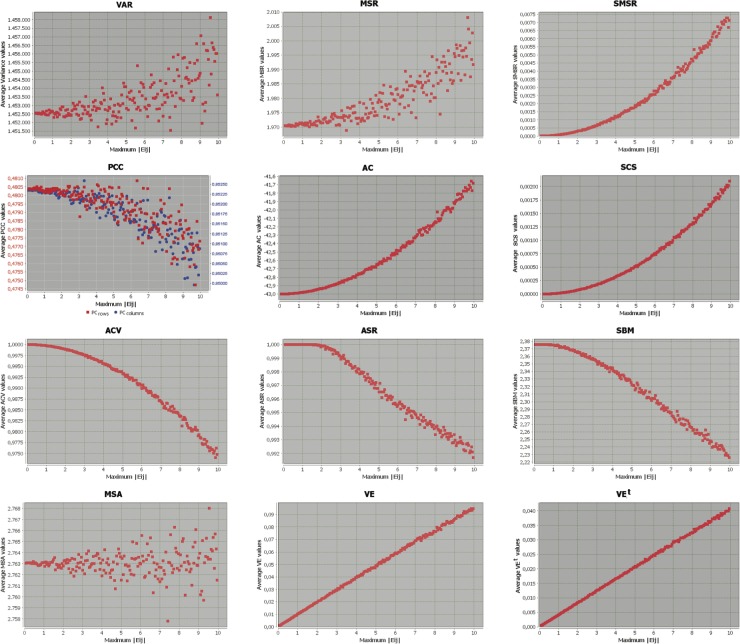
Evaluation measures evolution from a scaling base bicluster with induced errors.

**Figure 3:**
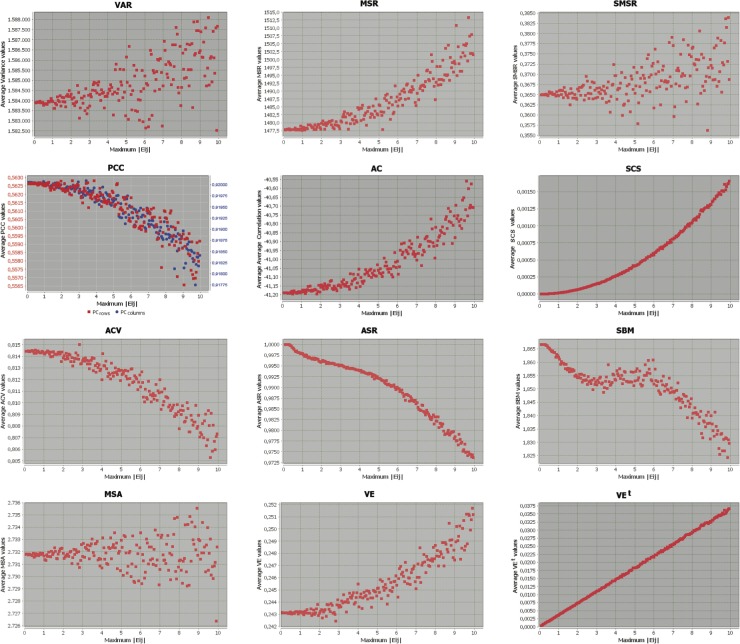
Evaluation measures evolution from a combined base bicluster with induced errors.

The results of these experiments are presented and analysed in the following subsections, where Figs. 1, 2 and 3 represent the behaviour of each metric when the maximum absolute error induced into the base bicluster varies from 0 to 10, as detailed previously. X-axes represent the absolute value of the maximum error induced (the amplitude of the range of errors is double its value), and the Y-axes correspond to the mean of the specific measure for each group of 100 biclusters. This way, the first value represented in the origin or the coordinate system corresponds to the evaluation of the base bicluster (without any induced error), while the last value on the right of the chart represents the evaluation value of the base bicluster with induced errors in their maximum possible amplitude (range [−10,10]). In all cases, the original interpretation of each metric has been retained, according to their description from their authors. This way, each evaluation measure has its corresponding range of values in the y-axis. Note also that only four out of the twelve metrics analysed need to be maximised (PCC, ACV, ASR and SBM), while for the rest of them their minimum value corresponds to the best evaluated bicluster. Finally, the first graph in the second row depicts the evolution of both row (gene) and column (condition) Pearsons Correlation Coefficients together, although in different ranges of values, represented in two Y-axes ranges.

### Biclusters with Shifting tendencies

Fig. 1 depicts the evolution of the different metrics when induced errors are incorporated into a base bicluster following a perfect shifting pattern. This way, the formula for the induced errors will be in the form of equation 31.

$$b_{ij}=\pi_{i}+\beta_{j}+\varepsilon_{ij}$$ (31)

The first row in Fig. 1 shows the evolution of Variance, MSR and SMSR. All of them display exponential behaviour regarding the amount of error, meaning that they are more permissive (or are not able to recognise) with lower amplitude errors. In the case of Variance, dots are more scattered on the graph, where it is possible to find that worse biclusters (with a larger amount of error) have been evaluated as better than biclusters closest to the perfect pattern. For example, for an amplitude equal to 14 (X-axis value equal to 7), several biclusters can be identified with a lower variance than some biclusters with error amplitudes between 10 and 12. This also holds true for SMSR, though it is not as pronounced. Note also that MSR reaches its minimum value for the base bicluster (0.0), while in the other two cases the perfect bicluster has not been evaluated with the minimum metric values.

The second and third rows in Fig. 1 correspond to the correlation based measures: PCC, Average Correlation, Correlation Score, ACV, ASR and SBM. Note that metrics based on Pearsons Correlation Coefficient present a logarithmic or exponential behaviour, depending on whether they need to be minimised or maximised for their optimum value, respectively. Regardless of their specific values, they can be considered as equally effective for detecting shifting trends in biclusters. Both ASR and SBM are based on Spearmans non-parametric coefficient, although they present different behaviours. ASR evaluates all biclusters with error amplitudes below 4 as perfect shifting biclusters, assigning to them its best possible value, equal to 1. It also presents a different behaviour in the maximum abs error ranges 2 to 4 and 4 to 10. The first range assimilates a linear progression, while the second one resembles a logarithmic progression. SBM, on the contrary, does not specify an optimal value for perfect trends. Its relationship with the amount of induced error could be considered to be linear, where evaluations are less disperse than for ASR.

The study of standardisation-based metrics (MSA, VE and VE^*t*^) is shown in the last row in Fig. 1. Although all of them will have lower values for better biclusters, MSA does not specify a minimum value, while VE and VE^*t*^ set their minimums at 0. Among them, Virtual Error (VE) is the one best at capturing the influences of error in a bicluster with a shifting pattern, since the representation of its evolution corresponds almost exactly to a linear progression, with very little dispersion between dots.

According to this study, the best linear behaviour for shifting trends in biclusters is best captured by Virtual Error (VE). However, Pearsons coefficient-based metrics, together with MSR, are better adapted for evaluating biclusters with shifting trends that display exponential or logarithmic behaviours.

### Biclusters with Scaling tendencies

Fig. 2 depicts the evolution of the different metrics when induced errors are incorporated into a base bicluster following a perfect scaling pattern. Hence, the formula for the induced errors will be in the form of equation 32.

$$b_{ij}=\pi_{i}\times \alpha_{j}+\varepsilon_{ij}$$ (32)

Although the majority of measures have an acceptable performance when recognising shifting trends in biclusters, fewer perform as well in the case of scaling patterns, as shown in Fig. 2. In particular, Variance, MSR, PCC (either on rows or columns) and MSA may not be used for this purpose, since all of them have evaluated a great number of biclusters with a larger amount of errors as better than the base biclusters that follow a perfect scaling pattern.

Nevertheless, although Pearsons coefficient (PCC) has been proven to be inefficient, its different based metrics seem to perform well under the measures Average Correlation, Correlation Score (exponential behaviour) or ACV (logarithmic behaviour). In the case or Spearmans based metrics, they are not able to recognise errors in biclusters when their amplitude is lower than 4 or 2, for ASR and SBM respectively. From these points, they present a quasi-linear tendency, with an acceptable dispersion level among the evaluations.

The standardised-based measures VE and VE^*t*^ in last row of Fig. 2 perform very well when detecting induced errors in biclusters with scaling trends, presenting an almost perfect linear behaviour, and adjusting the minimum possible metric value to the perfect base bicluster.

### Biclusters with Shifting and Scaling tendencies

Finally, Fig. 3 depicts the evolution of the different metrics when induced errors are incorporated into a base bicluster following a perfect combined pattern (including both shifting and scaling tendencies simultaneously). Hence, the formula for the induced errors will be in the form of equation 30, above. As shown in Fig. 3, only three out of the twelve original measures are efficient for evaluating biclusters with both shifting and scaling patterns. These metrics correspond to SCS (based on Pearsons coefficient and presenting an exponential tendency), ASR (based on Spearmans coefficient and presenting a logarithmic tendency from amplitude 4), and VE^*t*^ (based on standardisation and with linear behaviour).

### Performance Comparison with Real Datasets

This section examines the relations and dependencies among all the reviewed measures using biclusters obtained from four distinct real data sets. For this purpose, the biclusters generated by EvoBexpa in [32] have been used, where one hundred biclusters were found for each datasets.

Table 1 shows the details of the datasets used in this experimentation, including their sizes as well as references to their corresponding publications. Yeast dataset is the smallest, made up of 2884 genes and 17 samples, and represents one the most used dataset for comparison of biclustering techniques. In fact, it is considered as a benchmark dataset for many researches. Leukemia dataset is the one containing higher number of samples, while Steminal acts as the most unbalanced one, with the larger number of genes (26127) and only 30 samples. The diversity among dataset sizes and characteristics enriches the conclusions obtained from our study, extracting thus dependencies among measures for biclusters under different and varied peculiarities.

**Table 1:** Real Datasets used for Performance Comparison.

**Dataset**	**Name**	**#genes**	**#conditions**	**Ref.**
Yeast	Yeast *Saccharomyces cerevisiae* cell cycle	2884	17	[33]
Embryonal	Embryonal tumors of the central nervous syst.	7129	60	[34]
Leukemia	Leukemia	7129	72	[35]
Steminal	Steminal Cells	26127	30	[36]

The purpose of the study is to determine the extend to which each metric is related to one another, as well as assess their dependencies with the biological relevance of biclusters. In order to perform this second type of comparisons, there have been defined three metrics for measuring bicluster biological significance in terms of Gene Ontology (GO) [37] annotations.

Gene Ontology has been widely used in genome research applications, and also for the validation of results obtained after a microarray analysis process, such as clustering or biclustering. It is based on a graph hierarchy whose nodes represent terms dealing with molecular functions, cell components or biological processes, and edges connecting nodes depict dependency relationships between them. Term-for-Term analysis represents the standard method of performing statistical analysis for over-representation in GO. Starting from a subset of genes (study group) from a larger population (whole set of genes in the database), we are interested in knowing if the frequency of an annotation to a Gene Ontology term is relevant for the study group (genes in a bicluster), compared to the overall population. After this process and some necessary adjustments, a p-value is obtained for each term in GO for which genes in the study group are involved. Depending on the desired confidence level (p-value), a bicluster is said to be significantly enriched if there exists at least one GO term for which genes in the bicluster are significantly annotated.

The metrics included in the analysis are the following:

- **Best p-value (bestPValue)**. Corresponds to the p-value associated to the term annotated to a bicluster with a minimum p-value.
- **Mean of p-values (meanPValue)**. This value is obtained by computing the mean of all the p-values of the terms annotated to a bicluster.
- **Number of significant terms (numSigTerms)**. It is the number of significant terms annotated to a bicluster. A term is said to be significantly annotated if its p-value is less than or equal to a certain confidence level (0.05 has been used in this study).

In order to quantify the dependencies among the reviewed metrics, two different test cases were established. The first one includes all the biclusters found by Evo-Bexpa in [32] for all datasets. This constitutes a total amount of 400 biclusters (100 for each data set). The second test case only includes those biclusters that have been proven to be biologically significant, according to the former description, for each dataset. It comprises a total of 89 biclusters (41 from Yeast, 9 from Embryonal, 12 from Leukemia and 27 from Steminal). Pairwise comparisons among the values obtained from evaluating each bicluster with each metric were performed. This way, for the first test case, 120 comparisons have been carried out between 16 vectors of size 400, each vector containing 400 biclusters evaluations using a specific measure. The same procedure has been applied for the second test case, where the vectors contain 89 evaluation values.

The comparisons have been performed using *mutual information* (MI), a measure of association between variables that detects both linear and non-linear relationships, based on the use of probabilities [38]. MI constitutes a better alternative to the correlation coefficients of Pearson or Spearman, which only find linear dependencies. The application of MI is specially useful for studying complex biological systems, where its elements (functionally diverse and often multifunctional) are likely to interact in a non-linear way [39].

**Figure 4:**
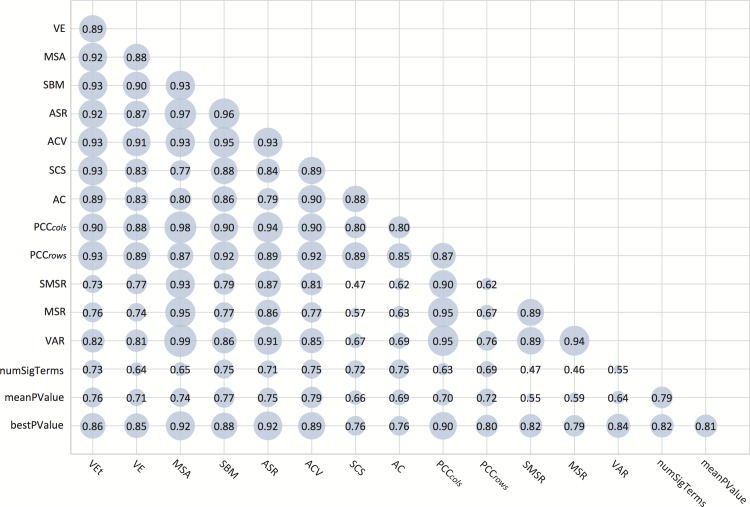
Mutual information dependencies among metrics for the first test case.

**Figure 5:**
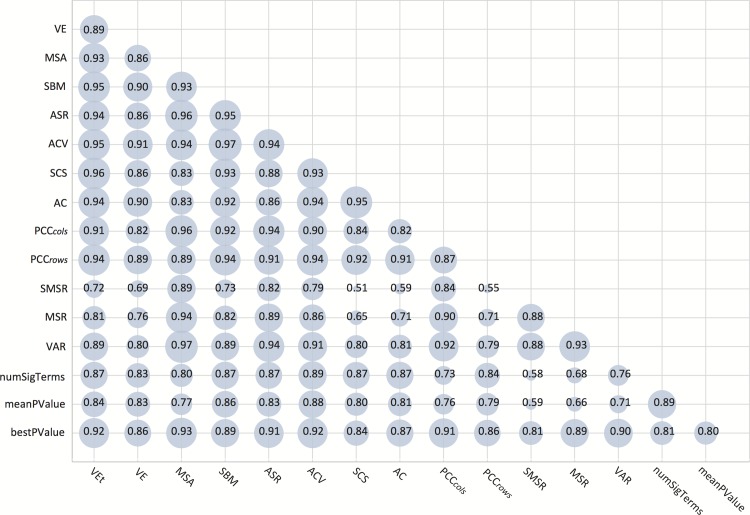
Mutual information dependencies among metrics for the second test case.

Figs. 4 and 5 represent the obtained comparison results between each pair of metrics reviewed in this work, together with the three GO-based metrics, for each test case. The area of the circles is proportional to the MI value between two specific metrics, where the higher the MI between two measures is, the more dependent they are, and where MI is zero if and only if the two variables are strictly independent. Nevertheless, MI is a non-standardised measure, meaning that when two variables are completely correlated their MI can be any value higher than 0. In order to estimate the dependency level between two variables, the Equation. 33 has been used, which standarises the MI value within the interval [0, 1]. To improve the visualization and make the dependencies clearer to the reader the minimun standardised MI value (0.4) corresponds to 0 in the Figure. This way, the area of each circle is between 0 (standardised MI of 0.4) and 1 (standardised MI of 1).

$$\mathrm{Standardised}\_\mathrm{MI}={\left(1-e^{-2*\mathrm{MI}}\right)}^{1∕2}$$ (33)

In both figures, the three first lines from the bottom depict the relationships between all reviewed measures and the three GO-based ones. Although in general all the metrics seem to be dependent on these three ones, it is the best p-value to which all of them are more related. This situation is more noticeable for the first test case, when using the whole set of 400 biclusters. Even when it was an expected result, this observation constitutes a good indicator for all measures, since both the number of significant terms and the mean of the p-values are more influenced by non significant biclusters in the first test case (Fig. 4) than the best p-value. Nevertheless, when assessing dependencies for the second test case (Fig. 5), where only significant biclusters were used, all metrics show high correlation levels with the GO-based ones. It can also be derived from Figs. 4 and 5 that all metrics are more dependent on each other for the second test case. This means that there exists a general tendency of agreement for all metrics when evaluating significant biclusters, bearing thus out their appropriateness for bicluster evaluation. Figs. 4 and 5 also include specific information on the level of dependency among each pair of measures, which has been numerically represented for each circle.

Tables 2 and 3 show a summarised ranking of the relationships among all measures for the two aforementioned test cases, respectively. Both tables present the three measures with highest MI for each metric (Ranking columns), including the three GO-based metrics (see the first three rows of the tables). Auto-dependencies have been omitted for these three measures, since these are not part of the reviewed metrics in this work. It can be observed that the measures ACV, MSA, SBM, VEt and AC are the best related to the GO-based metrics. Another interesting result is that correlation-based measures (section *Correlation-based Measures*) present highest dependencies with other metrics of the same group, since there always exists at least one correlation-based measure among the three ranking columns. This fact is even more evident for the significant biclusters in the second test case (Table 3), where each correlation-based metric presents highest values with at least two other measures in the same group. However, this fact does not occur with the standardisation-based group.

**Table 2:** Mutual Information based Ranking for the first test case.

**Measure**	**Ranking**		
	**1st**	**2nd**	**3rd**
bestPValue	MSA (0.924)	ASR (0.919)	PCC_*cols*_ (0.898)
meanPValue	ACV (0.786)	SBM (0.773)	VE*^t^* (0.761)
numSigTerms	ACV (0.748)	AC (0.747)	SBM (0.745)
			
VAR	MSA (0.985)	PCC_*cols*_ (0.953)	MSR (0.939)
MSR	PCC_*cols*_ (0.946)	MSA (0.946)	VAR (0.939)
SMSR	MSA (0.926)	PCC_*cols*_ (0.900)	VAR (0.894)
PCC_*rows*_	VE*^t^* (0.929)	ACV (0.923)	SBM (0.920)
PCC_cols_	MSA (0.977)	VAR (0.953)	MSR (0.946)
AC	ACV (0.899)	VE*^t^* (0.887)	SCS (0.877)
SCS	VEt (0.930)	ACV (0.892)	PCC_*rows*_ (0.885)
ACV	SBM (0.953)	VEt (0.934)	MSA (0.933)
ASR	MSA (0.969)	SBM (0.957)	PCC_*cols*_ (0.942)
SBM	ASR (0.957)	ACV (0.953)	MSA (0.932)
MSA	VAR (0.985)	PCC_*cols*_ (0.977)	ASR (0.969)
VE	ACV (0.906)	SBM (0.897)	VE*^t^* (0.893)
VE*^t^*	ACV (0.934)	SCS (0.930)	PCC_*rows*_ (0.929)

**Table 3:** Mutual Information based Ranking for the second test case.

**Measure**	**Ranking**		
	**1st**	**2nd**	**3rd**
bestPValue	MSA (0.932)	ACV (0.924)	VE*^t^* (0.922)
meanPValue	ACV (0.881)	SBM (0.861)	VE*^t^* (0.843)
numSigTerms	ACV (0.889)	SBM (0.874)	AC (0.873)
			
VAR	MSA (0.966)	ASR (0.936)	MSR (0.932)
MSR	MSA (0.941)	VAR (0.932)	PCC*_cols_* (0.901)
SMSR	MSA (0.891)	VAR (0.881)	MSR (0.880)
PCC_*rows*_	VE*^t^* (0.944)	ACV (0.941)	SBM (0.938)
PCC_*cols*_	MSA (0.956)	ASR (0.942)	SBM (0.918)
AC	SCS (0.952)	VE*^t^* (0.940)	ACV (0.940)
SCS	VE*^t^* (0.959)	AC (0.952)	ACV (0.929)
ACV	SBM (0.972)	VE*^t^* (0.950)	PCC_*rows*_ (0.941)
ASR	MSA (0.963)	SBM (0.945)	PCC_*cols*_ (0.942)
SBM	ACV (0.972)	VE*^t^* (0.949)	ASR (0.945)
MSA	VAR (0.966)	ASR (0.963)	PCC_*cols*_ (0.956)
VE	ACV (0.914)	SBM (0.904)	AC (0.900)
VE*^t^*	SCS (0.959)	ACV (0.950)	SBM (0.949)

## Summary

This paper studies and compares different measures for bicluster evaluation, both in an analytical and experimental way. It is important to note that all the measures presented in this study have been previously used within different biclustering search strategies, producing interesting and quality results that have also been biologically validated in most cases. Nevertheless, many authors have pointed out the impossibility of carrying out a fair comparison among the different biclustering approaches. This is due to the fact that the output biclusters may not be comparable in themselves, belonging to different biological categories.

The study presented here first reviewed the metrics under examination, grouping them according to their similar characteristics, and analysing their capacity to recognise different expression patterns in biclusters. Furthermore, in order to support and enhance this analysis, an experimental study was carried out using all the metrics to evaluate biclusters presenting induced errors with different amplitudes. For this purpose, biclusters were evaluated based on perfect shifting, scaling and combined patterns, also including negative correlations in all cases. These experiments allowed a graphical comparison to be performed between all metrics, evidencing their abilities to recognise the different kinds of patterns, together with their behaviour in the presence of various error amounts. These behaviours can be categorised into four main tendencies: exponential, logarithmic, linear and constant, the first two being conceptually equivalent, but depending on whether the corresponding measure needs to be maximised or minimised, respectively. Hence, exponential/logarithmic behaviours are more permissive with lower amplitude errors, while linear ones represent the same importance to all ranges. According to these categories, the best evaluation measures are reported for each type of expression pattern.

Table 4 outlines all the measures studied with their corresponding bibliographical references and a summary of the experimental results obtained. For each group of experiments (shifting, scaling and combined patterns), three indicators are shown: *Tendency*, which can take the values *Exp* (exponential), *Log* (logarithmic), *Lin* (linear), *Con* (constant), *NT* (non definite tendency); *Optimal*, which indicates whether the measure attains its optimal value when the term *ε_ij_* is zero or very low; and *Scatter*, which points out the scatter level of the measure values with regard to the growth of the *ε_ij_* term, and can take the values: *NS* (non scatter), ↓↓ (very low), ↓ (low), ↑ (high) and ↑↑ (very high).

**Table 4:** Summary of Bicluster Evaluation Measures.

**Measure**	**Ref.**	**Experimental Results**								
		**Shifting Patterns**			**Scaling Patterns**			**Combined Patterns**		
		**Tendency**	**Optimal**	**Scatter**	**Tendency**	**Optimal**	**Scatter**	**Tendency**	**Optimal**	**Scatter**
VAR	[11]	Exp		↓	Exp		↑	NT		↑↑
MSR	[12]	Exp	•	NS	Exp		↑	Exp		↑
SMSR	[18]	Exp		↓↓	Exp	•	↓↓	NT		↑↑
RI	[19]	-	-	-	-	-	-	-	-	-
PCC	[20, 21]	Log	•	NS	Log		↑	Log		↑
AC	[22]	Exp		NS	Exp		low	Exp		↑
SCS	[23]	Exp	•	NS	Exp		NS	Exp	•	NS
ACV	[24]	Log	•	NS	Log	•	NS	Log		↑
ASR	[26]	Cons-Lin-Log	•	↓↓	Cons-Lin	•	↓↓	Cons-Lin-Log	•	↓↓
SBM	[27]	Lin		NS	Cons-Lin		↓↓	Cons-Lin		↑↑
MSA	[28]	Exp		↓↓	NT		↑↑	NT		↑↑
VE	[29]	Lin	•	NS	Lin	•	NS	Exp		↑
VE*^t^*	[30]	Lin	•	NS	Lin	•	NS	Lin	•	NS
SS	[31]	-	-	-	-	-	-	-	-	-

Regardless of the type of tendency, the best measures are those that are capable of returning their optimal value for the base bicluster, together with a non-scatter behaviour. According to this table, six out of the twelve evaluated measures meet these standards for the shifting patterns, whereas only three and two of them meet these requirements for the shifting and combined patterns, respectively. Among all of them, VEt is the only measure that fulfils these requirements for all kind of patterns.

A performance assessment has also been carried out with biclusters obtained from real datasets. In this part of the study, reviewed metrics have been compared according to their evaluations for the biclusters in two groups: a first group made up of 400 obtained from four different datasets, and a second one containing only those significant biclusters in the first group (89 biclusters in total). The purpose of this study is to determine the extend to which each metric is related to one another, as well as assessing their dependencies with biclusters biological relevance, using Gene Ontology annotations. Results of these experiments are summarized in Figs. 4, 5 and Tables 2, 3, from which it can be derived that all metrics are more dependent on each other for the second test case, where only biclusters with significant GO annotations where used. Although this situation proves the appropriateness of all metrics, Tables 2 and 3 also point out the highest dependencies among them, where the 5 most related measures with the GO-based ones are MSA, ACV, SBM, VE^*t*^ and AC.

The availability of a suitable quality metric for biclusters is essential not only for guiding the search, but also for establishing comparison criteria among the results obtained by different biclustering techniques. Hence, the study presented here is deemed to be of considerable relevance in biclustering research. It may be used not only as a guide to select the proper measure for a biclustering-based experiment, but it can also contribute to this field of knowledge by taking an initial step toward solving the biclustering comparison problem.
